# Prodigiosin Modulates the Immune Response and Could Promote a Stable Atherosclerotic Lession in C57bl/6 *Ldlr*-/- Mice

**DOI:** 10.3390/ijms21176417

**Published:** 2020-09-03

**Authors:** Alejandro Cuevas, Nicolás Saavedra, Luis A. Salazar, Marcela F. Cavalcante, Jacqueline C. Silva, Dulcineia S. P. Abdalla

**Affiliations:** 1 Clinical Microbiology Unit, Department of Preclinical Sciences, Faculty of Medicine, Universidad de La Frontera, Manuel Montt 112, Temuco CP 4781176, Chile; 2 Centro de Investigación en Medicina de Laboratorio—CeMLab, Faculty of Medicine, Universidad de La Frontera, Manuel Montt 112, Temuco CP 4781176, Chile; nicolas.saavedra@ufrontera.cl; 3 Center of Molecular Biology and Pharmacogenetics, Scientific and Technological Bioresource Nucleus, Universidad de La Frontera, Francisco Salazar 01145, Temuco CP 4811230, Chile; luis.salazar@ufrontera.cl; 4 Department of Clinical and Toxicological Analysis, Faculty of Pharmaceutical Sciences, Universidade de São Paulo, São Paulo–SP 05508-000, Brazil; marcela.frotac@gmail.com (M.F.C.); jacque_cavalcante@yahoo.com.br (J.C.S.); dspa@usp.br (D.S.P.A.)

**Keywords:** prodigiosin, undecylprodigiosin, atherosclerosis, plaque stability

## Abstract

Atherosclerosis is a chronic inflammatory disease, whose progression and stability are modulated, among other factors, by an innate and adaptive immune response. Prodiginines are bacterial secondary metabolites with antiproliferative and immunomodulatory activities; however, their effect on the progression or vulnerability of atheromatous plaque has not been evaluated. This study assessed the therapeutic potential of prodigiosin and undecylprodigiosin on inflammatory marker expression and atherosclerosis. An in vitro and in vivo study was carried out. Migration, low-density lipoprotein (LDL) uptake and angiogenesis assays were performed on cell types involved in the pathophysiology of atherosclerosis. In addition, male LDL receptor null (*Ldlr*-/-) C57BL/6J mice were treated with prodigiosin or undecylprodigiosin for 28 days. Morphometric analysis of atherosclerotic plaques, gene expression of atherogenic factors in the aortic sinus and serum cytokine quantification were performed. The treatments applied had slight effects on the in vitro tests performed, highlighting the inhibitory effect on the migration of SMCs (smooth muscle cells). On the other hand, although no significant difference in atherosclerotic plaque progression was observed, gene expression of *IL-4* and *chemokine (C-C motif) ligand 2* (*Ccl2*) was downregulated. In addition, 50 µg/Kg/day of both treatments was sufficient to inhibit circulating tumor necrosis factor alpha (TNF-α), interleukin-2 (IL-2) and interferon-gamma (IFN-γ) in serum. These results suggested that prodigiosin and undecylprodigiosin modulated inflammatory markers and could have an impact in reducing atherosclerotic plaque vulnerability.

## 1. Introduction

Atherosclerosis is the pathophysiological process that underlies the development of the main cardiovascular diseases and is the leading cause of death in industrialized countries [1]. There is considerable evidence suggesting that atherosclerosis is more than just a lipid disorder, but also an inflammatory process involving the recruitment of many different cell types, including endothelial and smooth muscle cells, monocyte-derived macrophages and T lymphocytes [2]. Thus, atherosclerosis is currently defined as a chronic inflammatory disease, involving both innate and adaptive immune responses [2].

T cells, macrophages and smooth muscle cells (SMCs) are found in atherosclerotic plaques, and lead to the development of atherosclerosis by releasing cytokines, growth factors, and pro inflammatory mediators [3]. Although atherosclerosis progression has been mainly related to an inflammatory core of lipid-laden macrophages [4], T cell-mediated immune responses play an important but dual role in atherogenesis [5]. Several suspected self-antigens promote the differentiation of naïve T cells into effector T cells (Teffs), and may promote atherosclerotic disease [6]. Conversely, a protective role against atherosclerosis has been described for regulatory T cells (Treg) that express interleukin-2 receptor alpha chain (CD25) and the transcription element fork-head box P3 (FOXP3) by down-regulating inflammatory responses [5]. Consequently, there are data suggesting that shifting the Treg/Teff balance toward Tregs may be a possible therapeutic approach for atherosclerotic disease, although the role of Tregs in human atherosclerotic disease has not been fully elucidated [6].

Prodigiosin-like pigments or prodiginines (PDGs) are tripyrrole red-colored compounds produced as secondary metabolites by many terrestrial and marine bacterial strains, including species of *Serratia*, *Streptomyces*, *Hahella* and *Vibrio* [7,8]. These compounds have biological properties, including antitumor, immunosuppressing, and antimalarial activities at nontoxic levels [9,10]. Currently, most studies using PDGs relate to their antibiotic and cytotoxic effects; however, some studies have shown that compounds such us undecylprodigiosin (UPG) and prodigiosin (PG) selectively modulate T lymphocyte proliferation, acting on cytolytic T lymphocytes (CTLs) [11,12,13], and even modulating the response of macrophages to inflammatory stimuli [14]. For mouse splenic T lymphocytes stimulated with proinflammatory concanavalin A (ConA), PG efficiently blocked T cell activation by suppressing IL-2R formation and disrupting IL-2/IL-2R signaling, inhibiting the production of INF-γ and IL-4, but not affecting IL-2 secretion [15]. In addition, UPG induces the disorganization of cytotoxic granules and degradation of perforin, which results in inactivation of the cytotoxic function and a decrease in the number of activated mature CD8+ CTL in vitro and in vivo [16]. It was shown that PG reduces NO production and inducible nitric oxide synthase (iNOS) expression in murine peritoneal macrophages by inhibiting lipopolysaccharide (LPS)-triggered p38 mitogen-activated protein kinase (MAPK), c-Jun N-terminal kinase (JNK) phosphorylation and nuclear factor-kappa beta (NF-κB) activation [14]. Additionally, an in silico analysis performed to evaluate the anti-inflammatory activity against cyclooxygenase-2 (COX-2) protein showed that the PG ligand had a best fitness score comparable with the standard drug rofecoxib, suggesting that PG could be effective as a COX-2 inhibitor [17].

Innate and adaptive immunity has a key role in the induction and worsening of atherosclerotic lesions [18]. In this sense, PDGs could act on cells of both types of immunity and modulate the severity of atherosclerosis. T cell repression by PG would represent a double-edged sword in atherogenesis, as it could inhibit both pro-inflammatory T cell activation and atheroprotective FOXP3 (+) Treg responses by disruption of the IL-2/IL-2R alpha pathway. However, in advanced lesions, the resident T cells predominantly exhibit a type 1 T helper cell (Th1) phenotype secreting IFN-γ, which exacerbates lesion progression [19,20,21]. In addition, PG does not affect the production of IL-2, which would not affect the expansion of atheroprotective Tregs [22]. These findings suggest that PG could exert an immunomodulatory effect on T cells, which in turn might contribute to modifying the progression of atherosclerotic plaques. To test this hypothesis, the in vitro effect of these compounds on key processes in endothelial cells, smooth muscle and macrophages, three of the main cellular phenotypes involved in the pathophysiology of atherosclerosis, was evaluated. On the other hand, to evaluate possible effects on atherogenesis, inflammation and immune response, an in vivo trial was designed in C57bl/6 *Ldlr*-/- mice, which were induced with advanced atherosclerotic lesions before starting the respective treatments. The choice of this animal model was based on the need for a functional APOE protein, given the importance of this protein in modulating various inflammatory processes involved in atherogenesis [23]. As such, this study evaluated the effect of bacterial PG and UPG on inflammatory markers in cells involved with atherosclerosis, and their impact on atherosclerotic plaque vulnerability.

## 2. Results

### 2.1. Effect of PG or UPG on Viability and Cell Cycle of HUVEC (Human Umbilical Vascular Endothelial Cell), SMC and THP-1 Derived Macrophage-Like Cells

**Figure 1:**
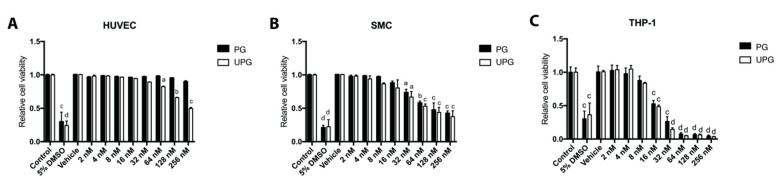
Effect of increasing concentrations of prodigiosin or undecylprodigiosin on HUVEC (human umbilical vascular endothelial cell) (**A**), SMC (smooth muscle cell) (**B**) and THP-1 derived macrophage-like (**C**) cell death as determined by annexin/PI staining and flow cytometry. HUVEC, SMC or THP-1 monolayers were treated with concentrations of PG (prodigiosin) or UPG (undecylprodigiosin) ranging from 2 nM to 256 nM for 24 h at 37 °C. 5% DMSO was included as a cell death control. Data are expressed as relative quantification of control groups (mean ± SD). ^a^, *p* < 0.05; ^b^, *p* < 0.01; ^c^, *p* < 0,001; ^d^, *p* < 0.0001; ANOVA with Dunnett’s post hoc test. The results were confirmed in three independent experiments (5 replicates per experiment).

An annexin V-FITC/PI staining assay was used to evaluate the viability of cellular lines exposed to different concentrations of PG or UPG (2–256 nM). Treatment with any concentration of PG or UPG up to 32 nM did not induce apoptosis or necrosis in HUVEC (human umbilical vascular endothelial cell) (Figure 1A). In addition, derived macrophage-like cells from both treatments up to 16 nM for SMC (Figure 1B) and up to 8 nM for THP-1 (Figure 1C) did not significantly decrease cell viability. On the other hand, no concentration of PG or UPG induced arrest of the cell cycle in any of the evaluated cell lines (data not shown). In order to work with non-toxic and non-apoptotic concentrations, the maximum concentration that did not alter the viability of the cells was selected for the subsequent experiments.

### 2.2. Effect of PG or UPG on Migration of HUVEC and SMC

**Figure 2:**
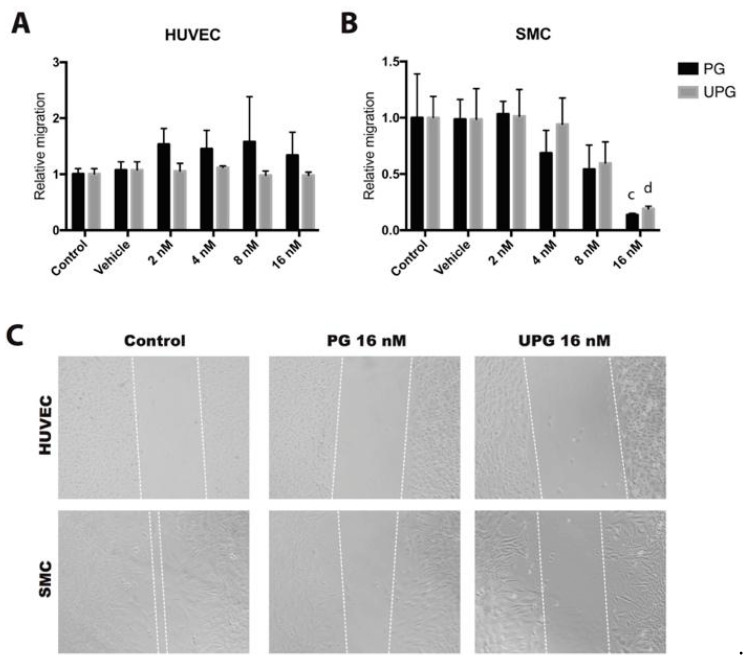
Effect of increasing concentrations of prodigiosin or undecylprodigiosin on cell migration by the scratch wound assay. HUVEC monolayers were incubated for 8 h with 10 ng/mL vascular endothelial growth factor A (VEGF-A), while SMC monolayers were incubated for 24 h with 30 ng/mL human platelet-derived growth factor-BB (PDGF-BB). Quantification of HUVEC (**A**) or SMC (**B**) migration relative to control group. Data are expressed as mean ± SD. ^c^, *p* < 0,001; ^d^, *p* < 0.0001; ANOVA with Dunnett’s post hoc test. (**C**) Representative pictures show cell migration after 24 h of treatment. Dashed lines flank non-invaded area. The results were confirmed in five independent experiments (5 replicates per experiment).

A scratch wound assay was performed to evaluate the possible inhibitory effect of PG or UPG on HUVEC and SMC migration. The treatments evaluated did not affect the in vitro migration of endothelial cells (Figure 2A). By contrast, increasing concentrations of PG and UPG showed an inhibitory effect on SMC migration (−86%, *p* < 0.001 and −81%, *p* < 0.0001, respectively), with significant differences at 16 nM (Figure 2B).

### 2.3. Effect of PG or UPG on In Vitro Angiogenesis

**Figure 3:**
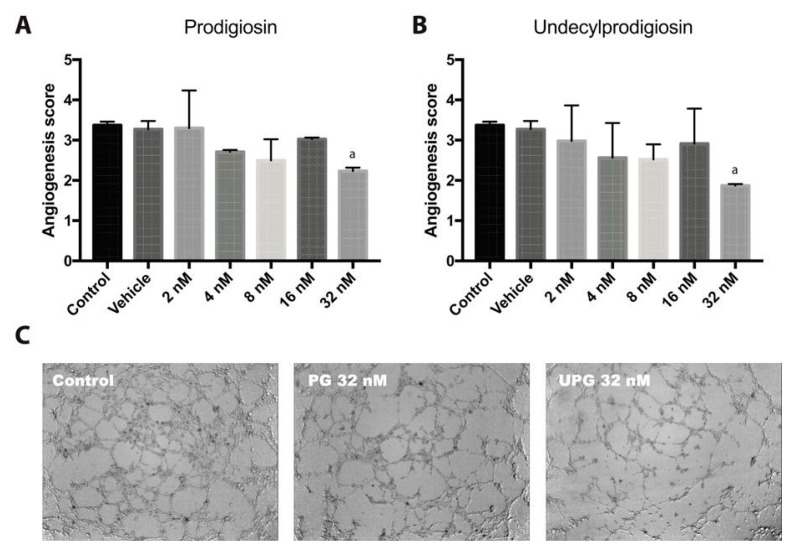
Tube formation of HUVECs using Matrigel basement membrane matrix. Different concentrations of PG or UPG were evaluated in the presence of 10 ng/mL VEGFA for 6 h at 37 °C in CO_2_ atmosphere_._ Graphical representation of tube formation for prodigiosin (**A**) and undecylprodigiosin (**B**). The angiogenesis score (scale without unit) was calculated considering the number of sprouting cells, connected cells and polygons, and the complexity of the formed mesh. Data are expressed as mean ± SD. ^a^, *p* < 0.05; ANOVA with Dunnett’s post hoc test. (**C**) Representative photographs after 6-h treatment with 32 nM prodigiosin and 32 nM undecylprodigiosin. The results were confirmed in three independent experiments (3 replicates per experiment).

The endothelial cells’ capacity to form capillary-like structures was evaluated by in vitro angiogenesis assay. PG and UPG at 32 nM were able to significantly reduce the angiogenesis score by 33.7% (*p* < 0.05) and 44.5% (*p* < 0.05), respectively (Figure 3), implying a significant reduction of the complexity and number of meshes and branches, a reduction in the number of nodes and an increase of non-associated structures.

### 2.4. Effect of PG or UPG on oxLDL and LDL(-) Uptake

**Figure 4:**
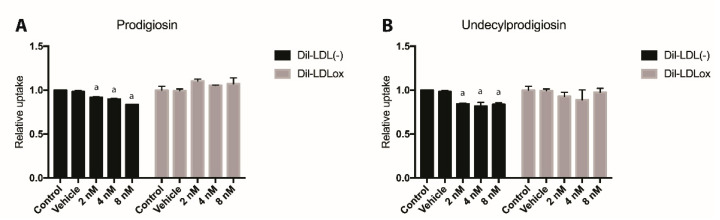
Dil-LDL(-) (electronegative LDL) or Dil-LDLox (labeled oxidized LDL) uptake by THP-1 derived macrophage-like cells. THP-1 macrophages were incubated with 50 µg/mL of labeled oxidized LDL (Dil-LDLox) or electronegative LDL (Dil-LDL(-)) at 37 °C for 24 h. Quantification of LDL (low-density lipoprotein) uptake relative to control group for prodigiosin (**A**) or undecylprodigiosin (**B**). ^a^, *p* < 0.05; ANOVA with Dunnett’s post hoc test. The results were confirmed in three independent experiments (5 replicates per experiment).

To assess the effect of PG on foam cell induction, the uptake of DIL labeled oxidized LDL (dil-oxLDL) and DIL labeled electronegative LDL (dil-LDL(-)) into THP-1 derived macrophage-like cells was quantified by flow cytometry. None of the treatments modified the uptake of oxLDL (Figure 4); however, both PG and UPG had a small but statistically significant effect on the uptake of LDL(-), reducing the uptake of LDL(-) up to 17% (*p* < 0.01), when treating cells with 8 nM PG or 8 nM UPG.

### 2.5. Effects of PDGs on Food Consumption, Animal Growth and Lipid Profile

**Figure 5:**
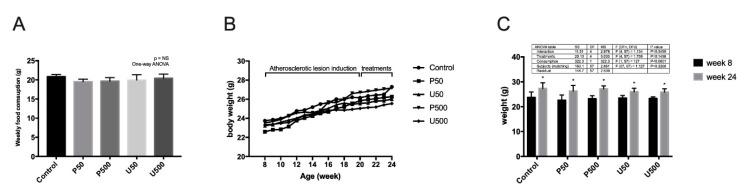
Weekly food consumption and weekly body weight of *Ldlr*-/- mice treated with prodigiosin or undecylprodigiosin. (**A**) Average weekly feed consumption per mouse colony was quantified (3 or 4 cages per group). The data are presented as the mean ± SD. No significant differences were observed. (**B**) Average weekly body weight in grams (growth chart) for all individuals per group, between 8 and 24 weeks of age. (**C**) Average body weight at 8 weeks of age, compared to average body weight at 24 weeks of age, for each group studied. It was observed that the treatments did not affect food consumption, nor did they influence the body weight of the animals. Eating food independently increased the body weight of the animals (*p* < 0.01, two-way ANOVA. *, *p* < 0.001 Sidak’s multiple comparison test for animal weight (g) between week 8 and week 24).

There were no significant differences in serum triglyceride, total cholesterol, HDL (high-density lipoprotein) cholesterol or LDL (low-density lipoprotein) cholesterol levels between treated mice and the control groups (Table 1). In addition, no significant changes were observed in the weekly food consumption between groups (Figure 5A). On the other hand, it was observed that the treatment with prodigiosine or undecylprodigiosine had no influence on the increase in body weight. This variable was influenced exclusively by feed consumption, showing statistically significant changes when comparing the average weights between the start and end weeks of the animal experiments (Figure 5B,C).

### 2.6. PDGs Do not Modify the Size of Atherosclerotic Lesions

**Figure 6:**
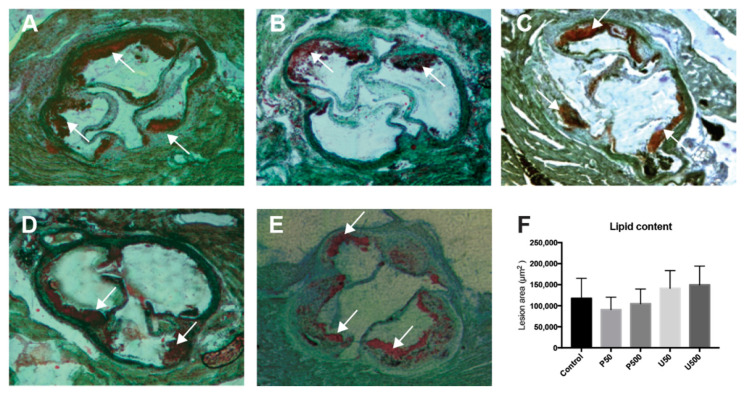
Atherosclerotic lesion sizes in *Ldlr*-/- mice treated with prodigiosin or undecylprodigiosin. Representative Oil Red O-stained atherosclerotic lesions for control (**A**) and P50 (**B**), P500 (**C**), U50 (**D**) and U500 (**E**) treatments. (**F**) Area of atherosclerotic lesions for controls and treated animals, presented as the mean ± SD (*n* = 7). No significant differences were observed.

The formation of atherosclerotic plaques was visualized by quantifying lipid content in sections of aortic sinus obtained from *Ldlr* knockout mice, using Oil Red O staining, demonstrating the validity of the model. Quantitative analysis of atherosclerotic lesion size indicated that the progression of aortic sinus injury was not modified in treated mice compared with control mice (Figure 6).

### 2.7. PDGs Modify the Expression of Genes Associated with Plaque Stability and Inflammatory Processes in the Aortic Sinus

**Figure 7:**
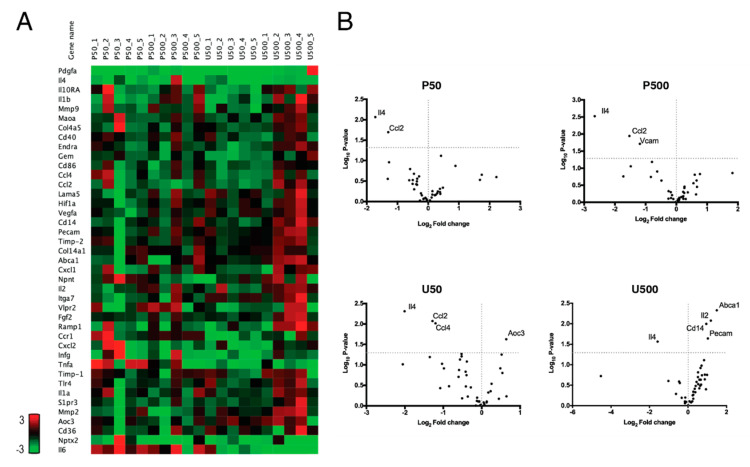
PCR array expression of genes associated with plaque stability and inflammatory processes in the aortic sinus of C57BL/6J *Ldlr*-/- mice treated for 28 days with PG or UPG. Heat map (**A**) and volcano plot (**B**) of genes altered by prodigiosin or undecylprodigiosin in the aortic sinus. Statistically significant gene expression can be observed in the upper left and right quadrants. Gene expression was quantified in the aortic sinus of 8 individuals per group.

The expression of a set of genes, including genes associated with inflammatory processes and plaque stability, was assessed by real time PCR. The set of evaluated genes did not show major differences among the different groups (Figure 7A); however, the U500 mice group showed an increased expression of the majority of the evaluated genes, in comparison to the other groups. Volcano plots (Figure 7B) summarized the differential expression of the evaluated genes, with statistically significant differences. All doses of PG and UPG were able to significantly repress the gene expression of *Il-4*. In addition, in the P50, P500 and U50 groups, the expression of *chemokine (C-C motif) ligand 2* (*Ccl2*) was slightly repressed. Furthermore, *vascular cell adhesion molecule 1* (*Vcam-1*) had a slight decrease in group P500, while the U50 group showed a slight decrease in the expression of *Ccl4* and an increased expression of *amine oxidase, copper containing 3* (*Aoc3*). Finally, expression of *platelet endothelial cell adhesion molecule* (*Pecam-1*), *Cd14*, *Il-2* and *ATP-binding cassette transporter A* (*Abca1*) was increased in the U500 group.

### 2.8. Effect of PG and UPG on Signature Transcripts of Different Th Cell Subsets from Paraaortic Lymph Nodes

**Figure 8:**
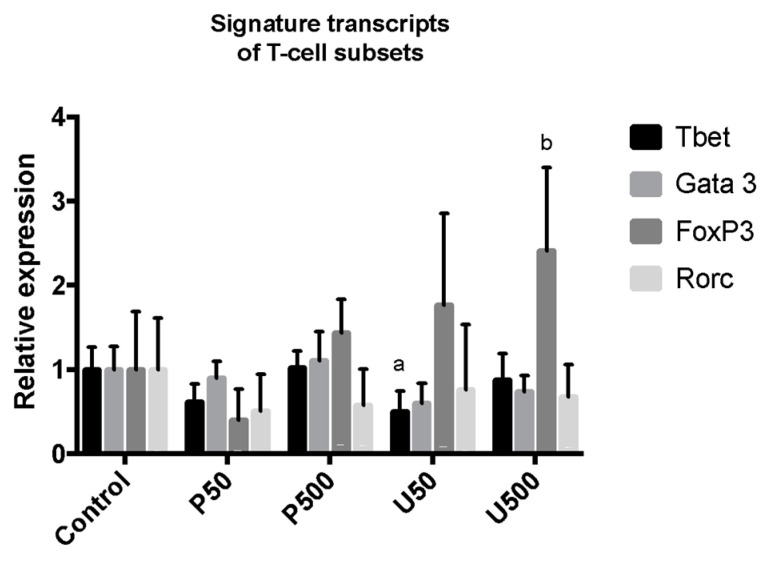
Relative gene expression of transcript factors of different Th cell subsets in paraaortic lymph nodes of C57BL/6J *Ldlr*-/- mice treated for 28 days with PG or UPG. ^a^, *p* < 0.05; ^b^, *p* < 0.01; ANOVA with Dunnett’s post hoc test. Gene expression was quantified in the paraaortic lymph nodes of 8 individuals per group.

In order to obtain a complementary measurement about the activation of T cells in lymphoid tissues that interact with the aortic tissue, the signature transcripts of different Th cell subsets in paraaortic lymph nodes were assessed by RT-PCR analysis of mRNA (Figure 8). No changes were observed in the expression of transcription factors *Gata3* (Th-2 marker) and *Rorc* (Th-17 marker). By contrast, *T-bet* (Th-1 marker) expression was attenuated in the U50 group. In addition, *Foxp3* (Treg marker) expression was overexpressed in the U500 group.

### 2.9. Effect of PG and UPG on Th1- and Th2-Related Cytokines in Serum

**Figure 9:**
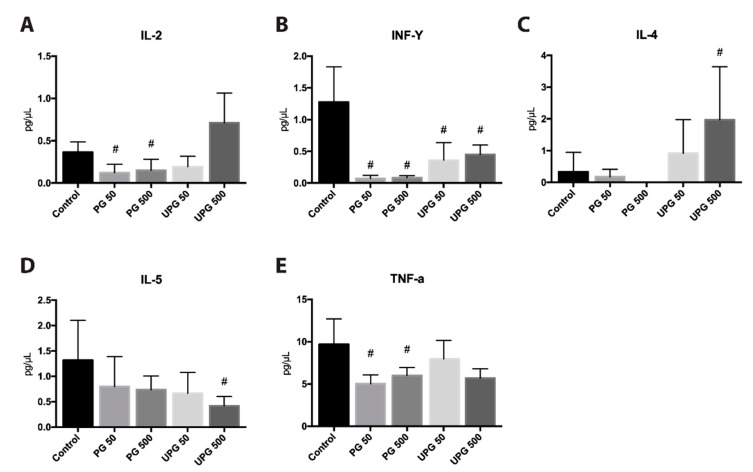
Quantification by BD™ Mouse Th1/Th2 Cytokine Cytometric Bead Array (CBA) of serum cytokines from C57BL/6 *Ldl*-/- mice treated with prodigiosin or undecylprodigiosin for 28 days. Cytokine levels of IL-2 (**A**), IFN-γ (**B**), IL-4 (**C**), IL-5 (**D**) and TNF-α (**E**). ^#^, *p* < 0.05; ANOVA with Dunnett’s post hoc test. Cytokine levels were quantified in the paraaortic lymph nodes of all individuals of each group.

Figure 9 shows the effects of PG or UPG on cytokines related with Th1 and Th2 lymphocytes. PG, independent of the dose, inhibited the production of TNF-α, IL-2 and IFN-γ. Similarly, UPG had a suppressive effect on IFN-γ, but not on Th1-related cytokines. On the other hand, PG had no statistically significant effects on Th2-related cytokines; however, it was not possible to detect IL-4 in the group treated with 500 µg/Kg/day PG. Treatment with 50 µg/Kg/day UPG did not induce any significant change in the concentration of IL-4 or IL-5, while treatment with 500 µg/Kg/day caused the overexpression of IL-4 and the inhibition of IL-5.

## 3. Discussion

PDGs and their derivatives are effective proapoptotic agents against various cancer cell lines, with multiple cellular targets, including multi-drug resistant cells. They show little or no toxicity towards normal cell lines; however, few studies have reported their effects on in vivo models. In the present study, possible in vivo anti-atherosclerotic effects of bacterial PDGs were evaluated for the first time.

Atherosclerosis is the underlying cause of acute coronary syndrome, myocardial infarction and stroke, and has become the leading cause of death and disability worldwide [24]. The rupture of vulnerable atherosclerotic plaques is the primary cause of coronary thrombosis and subsequent myocardial infarction. Although none of the treatments in our study had a significant effect on the progression of atherosclerotic lesions in mice, our results suggested that PG could have an impact on reducing plaque vulnerability.

In order to obtain an approximation of the effect of PG on the cell types that participate in the pathophysiology of atherosclerosis, cell cultures were examined. Our results showed a slight effect on LDL(-) uptake by macrophages with both treatments; however, no effect on oxLDL uptake was observed. This was consistent with LDL(-) and oxLDL having different affinities for scavenger receptors. In vitro studies have shown that macrophages internalize and degrade oxLDL principally by class B (SRBI, CD36) and class E (LDL receptor-1, LOX-1) scavenger receptors [25]. By contrast, LDL(-) has a conformational modification in the Apo B-100 lipoprotein, making it difficult to be recognized by these receptors [26] instead of being captured in an alternative manner, such as by CD14 and TLR4 [27,28]. In this regard, it has been demonstrated that PG is able to recognize and inhibit the Akt/mTOR pathway in melanoma cells [29]. This is relevant because it has also been shown that mTOR is involved in THP-1 macrophage-like foam cell formation, upregulating the expression of TLR4 [30]. This possible mTOR pathway inhibition could partly explain the observed effects. On the other hand, our results showed that PG and UPG slightly interfered in the formation of capillary-like structures. Similarly, the Akt/mTOR pathway is known to play a key role in increasing VEGF secretion and other proangiogenic factors, such as nitric oxide and angiopoietins [31]. Therefore, PDGs might partially inhibit in vitro angiogenesis through the inhibition of mTOR; however, further study of the mTOR signaling pathway would be necessary to corroborate these findings.

Other relevant processes such as monocyte transmigration at the beginning of atherogenesis could be evaluated in vitro, however, they were omitted from this research under the hypothesis that the evaluated treatments would have an effect on adaptive immunity, therefore it would be expected that the effects on the atherosclerotic lesion were late.

A strong inhibitory effect on the migration of SMCs in vitro was observed by both treatments. It is difficult to apportion the implications of this result, because the classical view of SMCs in atherosclerosis is that aberrant proliferation of these cells promotes plaque formation, while in advanced plaques they are entirely beneficial, for example, in preventing rupture of the fibrous cap [32].

It is necessary to consider that the cell types used are not the most representative models to explain the events that occurred in an atherosclerotic lesion, so these results should be interpreted with care. HUVEC is a widely used model in in vitro angiogenesis studies [33], however, they are not cells derived from arteries and these are derived from a large vessel, whereas angiogenesis occurs in microvessels. On the other hand, experiments performed with immortalized cells such as A10 (SMC) or THP-1 should be considered as simplified models when relatively simple biological processes such as cell migration or LDL uptake performed in this study are investigated, but not as a valid model for more complex studies [34].

In the in vivo model, we observed that PG and UPG were able to reduce serum levels of a set of immune cytokines that could impact powerfully on the fibrous cap. It is highlighted, for example, that both treatments were able to reduce the serum levels of IFN-γ, a pro-inflammatory, macrophage-activating cytokine produced by Th1-type T cells and natural killer (NK) cells. IFN-γ contributes to plaque, adopting a vulnerable phenotype with a reduced collagen content by inhibition of SMC infiltration, proliferation, and procollagen-I and III gene expression, and also by increasing the production of MMPs [35,36,37]. In addition, PG succeeded in reducing circulating IL-2 and TNF-α levels, two key players in modulating chronic immune responses by promoting leukocyte recruitment and polarizing T cells [3]. The reduction in TNF-α seemed especially relevant, because this cytokine induces the expression of matrix metalloproteinases that degrade collagen and promote tissue remodeling [38]. Related with this process, it has been observed that TNF-α plays a key role in the development of atherosclerosis [39], being predominately expressed in early atherosclerotic lesions [40]. In addition, previous reports have shown that the concentration of TNF-α was higher in the serum of patients with atherosclerosis [41], indicating a relevance of these findings in the context of this pathology.

The reduction of IFN-γ could be related to an attenuation of the Th1 response, favoring a Th2 response. We observed a downregulation of IL-2 and IFN-γ levels in the serum of mice treated with PG, and downregulation of IFN-γ (in serum) and Tbet transcription factor (paraaortic lymph nodes) in those treated with UPG. In addition, IL-4 levels were increased in mice treated with UPG; however, IL-5 levels were attenuated. While the T-helper cell type 1 (Th1) response has a potent proatherogenic effect, the pathogenic roles of other T cell subsets, such as the Th2 and Th17 pathways, remain controversial. Even so, the antiatherosclerotic protective roles of Treg and some Th2-related cytokines, such as IL-5, have been clearly established [42]. Thus, our data suggested a protective effect of PDGs by attenuation of Th1 responses; however, the effect on IL-5 expression was contradictory.

When the expression of different anti and pro-atherogenic factors were studied in atherosclerotic lesions, it was interesting to note that regardless of the dose of PG or UPG, the gene expression of *Il-4* was downregulated. IL-4 is an immunomodulatory cytokine secreted by Th2 cells, and has traditionally been considered an anti-inflammatory cytokine [43]. However, a growing body of evidence has suggested that IL-4 is pro-atherogenic, and may play a critical role in the progression of atherosclerosis. For instance, it has been shown that IL-4 induces pro-inflammatory environments by overexpressing a number of pro-inflammatory mediators, such as VCAM-1, E-selectin, C-C motif chemokine 2 (monocyte chemoattractant protein-1, MCP-1), and IL-6 in human vascular endothelial cells [44,45]. Similarly, *Ccl2* mRNA expression was downregulated, except by the high dose of UPG. C-C motif chemokine 2 is a strongly expressed chemokine in macrophage-rich regions of human atherosclerotic lesions. Its role is vital in the instability and subsequent rupture of atheromatous plaques [46,47]. In this regard, a low dose of PG achieved downregulation of the gene expression of *Il-4*, *Ccl2* and *Vcam*, consistent with previously published results, demonstrating how beneficially PDGs could inhibit factors that could alter the stability of the atheroma plaque. By contrast, UPG induced the overexpression of some genes, such as *Aoc3*, *Pecam* and *Il-2*, which was not consistent with this hypothesis. It was observed that an increase in serum concentrations of vascular adhesion protein-1 (VAP-1), a member of the copper-containing amine oxidase/semicarbazide-sensitive amine oxidase (AOC/SSAO) enzyme family, can be considered a sign of preclinical atherosclerosis [48]. It has also been shown that intraperitoneal injection of IL-2 into ApoE−/−mice fed with a high-fat diet enhances atherosclerosis, while IL-2 antibody treatment has a protective effect, indicating that IL-2 is atherogenic [49].

It has been shown that first-line treatment with statins reduces LDL-C and, consequently, the risk of cardiovascular disease (CVD). Despite the increase in the use of statin monotherapy in the last decade, not all people achieve an optimal reduction of LDL-C [50]. Given this difficulty, the search for adjuvant therapies through the modulation of other targets is a relevant area of study. For example, in the sense of our study, it was recently described that inhibition of PCSK3 modulates MMP2 activity and attenuates inflammation, which could contribute to the atheroprotection observed in mice [51].

In summary, while our results showed an ambiguous effect for UPG, PG showed an effect on inflammatory and atherogenic markers that could be related to the induction of stable atherosclerotic plaque. In this context, PG might complement current treatments of atherosclerosis in advanced stages of lesions; however, additional studies need to be carried out to further refine the conclusions.

## 4. Materials and Methods

### 4.1. Cell Culture

Human umbilical vein endothelial cells (HUVECs) and the human monocytic cell line THP-1 were maintained in Roswell Park Memorial Institute (RPMI) 1640 medium (Gibco; Thermo Fisher Scientific, Inc., Waltham, MA, USA)), while the A-10 line of SMCs (ATCC CRL-1476) was maintained in Dulbecco’s modified Eagle’s medium (DMEM; Gibco). Both media were supplemented with 10% heat-inactivated fetal bovine serum (FBS), 2.0 g/L sodium bicarbonate, 16.5 mmol/L HEPES, 100 IU/mL penicillin and 100 μg/mL streptomycin. Cell cultures were maintained at 37 °C in a 5% CO_2_ humidified atmosphere. Prior to each experiment, THP-1 monocytes were exposed to 160 nM phorbol-12-myristate-13-acetate (Sigma-Aldrich, St. Louis, MO, USA) for 72 h, in order to differentiate them into macrophages. THP-1 macrophages differentiated by PMA were selected by removing nonattached cells by aspiration and subsequently washing with RPMI 1640, thus maintaining only the cells adherent to the plate.

### 4.2. Annexin V-Fluorescein Isothiocyanate (FITC)/Propidium Iodide (PI) Staining Assay

The viability of the cells exposed to different treatments was measured with an Annexin V-FITC Apoptosis Detection Kit (Sigma-Aldrich, St. Louis, MO, USA), according to the manufacturer’s protocol. Briefly, confluent HUVEC, SMC or THP-1 monolayers were treated with concentrations of PG or UPG ranging from 2 nM to 256 nM for 24 h at 37 °C. In addition, a 5% DMSO treatment was included as a control of cell death. Cells were then harvested, resuspended in 1×-binding buffer and stained with 10 μL Annexin V-FITC and 5 μL propidium iodide (PI) for 15 min at room temperature in the dark. Finally, the cell suspensions were analyzed on a BD^TM^ FACSCanto II flow cytometry system (Becton Dickinson, San Jose, CA, USA) to identify the subpopulations of apoptotic and necrotic cells.

### 4.3. Cell Cycle Analysis

The ratio of cells in the G0/G1, S, and G2/M phases of the cell cycle was determined by their DNA content. In 6-well plates, 2 × 10^5^ cells per well were treated with increasing concentrations of PG or UPG for 24 h. Cells were then harvested, transferred to cytometry tubes, and centrifuged. The cell pellets were resuspended in 200 μL lysis buffer (0.1% sodium citrate, 0.1% Triton), 20 μL RNAse A (Invitrogen^TM^, Thermo Fisher Scientific, Inc., Waltham, MA, USA), and 2 μL 1 mg/mL propidium iodide (Sigma-Aldrich, St. Louis, MO, USA). The cells were then incubated for 30 min at 37 °C and analyzed by flow cytometry.

### 4.4. Migration Assay

HUVEC and SMC migration was analyzed using an in vitro scratch wound assay, as previously described [52]. In brief, confluent HUVEC or SMC monolayers were scratched with a sterile pipette tip and rinsed. The HUVEC monolayers were incubated for 8 h with RPMI 1640 medium supplemented with 1% FBS and 10 ng/mL vascular endothelial growth factor A (VEGFA), while the SMC monolayers were incubated for 24 h with DMEM supplemented with 1% FBS plus and 30 ng/mL human platelet-derived growth factor-BB (PDGF-BB). After incubation, the wounding areas were measured and analyzed using TScratch software [53]. In order to determine the extent of migration, three distinct representative microscopic fields of each culture plate were measured. Each experiment was carried out in triplicate, and each experiment was repeated three times.

### 4.5. Tube Formation Assay

The capillary-like formation assay was performed as previously described [54], with slight modifications. An In Vitro Angiogenesis Assay Kit (Merck Millipore, Burlington, MA, USA) was thawed at 4 °C overnight. Fifty μL of ECMatrix™ was added to each well of a 96-well culture plate and was allowed to polymerize at 37 °C for 30 min. The HUVECs, to be tested for tube formation, were detached from the tissue culture plates, washed, and resuspended at 8 × 10^3^ cells/well in RPMI 1640 medium containing 1% FBS. They were then added to the ECMatrix™-coated wells with different concentrations of PG or UPG in the presence of 10 ng/mL VEGFA. The plates were incubated at 37 °C for 6 h in 5% CO_2_. After incubation, capillary-like tube formation of each well in the culture plates was photographed using an Olympus light microscope. Each experiment was repeated three times, with two replicates performed each time. The angiogenesis score was calculated based on the number of sprouting cells, connected cells and polygons, and the complexity of the formed mesh, according to the formula described by Aranda and Owen [55].

### 4.6. DiI-OxLDL/Dil-LDL(-) Uptake Assays

THP-1 macrophages were incubated with 50 µg/mL oxidized LDL (oxLDL) or electronegative LDL (LDL(-)), labeled with modified 1,19-dioctadecyl-3,3,39,39-tetramethylindocarbocyane perchlorate (DiI), in RPMI 1640 containing 10% FBS at 37 °C for 24 h. After incubation, DiI-oxLDL and Dil-LDL(-) uptake was analyzed by flow cytometry.

### 4.7. Animals and Treatments

Seventy-five male LDL receptor null (*Ldlr*-/-) C57BL/6J mice, at 8 weeks of age, were obtained from the Laboratory of Production and Experimentation FCF-IQ, University of São Paulo, Brazil. The progenitors were purchased from the Jackson Laboratory (Bar Harbor, ME, USA). The total number of animals was estimated according to The Laboratory Animal Services Centre of The Chinese University of Hong Kong sample size calculator [56], considering a statistical power of 90%, an alpha level of 0.05 and an expected difference in the means of two compared groups (including standard deviations of each group) obtained from previous studies carried out in our laboratory. The experiments considered for this calculation were the quantitative analysis of atherosclerotic lesion and qPCR, because they use the entire aortic sinus tissue in their execution. Regardless of the estimated sample size to achieve the desired statistical power, a minimum of 3 and maximum of 15 animals per group was established. Thus, the calculation of the sample size resulted (for a statistical power of 80 to 90%) in a total of 7 to 9 individuals for qPCR and 5 to 7 individuals for morphometry/IHC of the atherosclerotic lesion, so it was decided to adjust the number per group to a total of 15 individuals.

The mice were received in a conditioned room of the Laboratory of Production and Experimentation FCF-IQ and were kept in groups of 3 to 5 individuals of the same litter (exceptionally 2 individuals) in polycarbonate cages for experimental rodents with gravel. At the time of receiving the animals, they showed normal appearance and behavior, no relevant clinical signs, with water and food consumption within the expected range and an average baseline weight of 23.31 ± 1.6 g. During the development of the experimental protocol, the animals received food and water ad libitum, and were maintained under a 12-h light/dark cycle, with controlled humidity and temperature conditions.

In the next 12 weeks, the animals were fed a 0.5% cholesterol-enriched hypercholesterolemic diet (HCD) and were monitored using an animal surveillance protocol [57], which allowed us to calculate a score by assessing appearance, water and food intake, weight loss, temperature, respiratory/cardiac frequency and behavior. When an alteration of the state of health or signs of suffering were observed, the animal was excluded from the study. In this regard, one animal presented an impaired state of health, so it was euthanized by carbon dioxide. At week 12 (20 week old mice), the mice were randomly divided into five experimental groups (*n* = 15) using GraphPad QuickCalcs Website [58]. Thus, each experimental group received the HCD plus a daily dose of a specific treatment: vehicle (0.9% NaCl, 0.25% Tween 80, 20% polyethylene glycol; control group); 50 µg/Kg/day PG (P50); 500 µg/Kg/day PG (P500); 50 µg/Kg/day UPG (U50); or 500 µg/Kg/day UPG (U500). The vehicle or treatments were administered by gavage for 28 days.

At the end of the treatment period, the weight of each mouse was recorded for the last time (average final weight 26.45 ± 1.9 g) and the euthanasia procedure was carried out. First, the animals were anesthetized with a mixture of ketamine hydrochloride 100 mg/kg + xylazine hydrochloride 20 mg/kg intraperitoneally administered and it was confirmed that the animal did not respond to stimuli in its limbs before continuing with the procedure. Then, a dissection was performed to quickly reach the thoracic cavity and obtain a blood sample by cardiac puncture (1 mL). In this way, the death of the animal was induced by exsanguination (acceptable method for unconscious animals). Finally, periaortic lymph nodes and cardiac tissues were collected and stored in their respective conservation media, pending further processing.

The protocol of this study (for all the animals used and the interventions performed) was approved by the Commission of Ethics in Animal Experimentation of the Faculty of Pharmaceutical Sciences, University of São Paulo, Sao Paulo, Brazil (protocol CEUA/FCF/392, 18 February 2013), to be in accordance with the rules of the National Council for Control of Animal Experimentation (CONCEA) and the Scientific Ethics Committee of Universidad de La Frontera (protocol 063/2014, 18 December 2014), Temuco, Chile, to comply with the guidelines on bioethical aspects of animal experimentation, of the Advisory Council of Bioethics, FONDECYT-CONICYT.

### 4.8. Serum Lipid Profile Analysis

Serum was obtained from blood samples of all animals included in the study (*n* = 75) and total cholesterol, triglycerides, high-density lipoprotein (HDL) cholesterol and LDL cholesterol was measured using colorimetric assays (Labtest Diagnostic SA, Lagoa Santa, MG, Brazil), according to the manufacturer’s protocol.

### 4.9. Morphometric Analysis of Atherosclerotic Plaques

Immediately after having obtained the tissues for analysis, the heart was perfused with phosphate buffered saline (PBS) and 10% PBS-buffered formaldehyde. Then, the tissue was fixed in 10% formaldehyde for at least 2 days and then embedded in 5%, 10% and 25% gelatin and specimen matrix for cryostat sectioning (Tissue-Tek^®^ O.C.T. Compound; Sakura^®^ Finetek, Torrance, CA, USA). The aortic sinus at the heart was sectioned proximally to distally in 1-μm-thick slices starting from the semilunar valves and then stained using Oil Red O to quantify atherosclerotic lesion areas. The area of the atherosclerotic lesion was reported as the average of the lesions in ten equidistant sections (40–50 μm) along an aortic sinus length of approximately 400 μm. The results from the seven mice per group are reported as mean of µm^2^/section ± S.D. Images from stained slides were obtained using an optical microscope equipped with a Nikon DXM 1200C digital camera and Nikon ACT-1C software. The quantification of atherosclerotic lesions followed guidelines previously used [59], and ImageJ 1.51p software (National Institutes of Health, Bethesda, MD, USA) was used to assess the captured images.

### 4.10. Gene Expression Analysis

A set of 44 genes were assessed in order to obtain an RNA expression signature associated with pathological features of vulnerable atherosclerotic plaque, as previously described [60]. In addition, gene expression of specific master transcription factors of Th-1 (*T-bet*), Th-2 (*Gata3*), Th-17 (*Rorc*) and Treg (*FoxP3*) lymphocytes was assessed in order to obtain signature profiles of different T-cell subsets from paraaortic lymph nodes.

Total RNA was isolated using TRI Reagent^TM^ Solution (Invitrogen^TM^, Thermo Fisher Scientific, Inc., Waltham, MA, USA). The quantification assays were performed by real-time reverse transcription polymerase chain reactions (RT-PCR). One µg total RNA was reverse-transcribed using High Capacity RNA-to-cDNA Master Mix (Applied Biosystems Inc., Foster City, CA, USA). Amplification reactions were performed in a Step-one Real-Time PCR System (Applied Biosystems Inc., USA) using an in-house PCR protocol. Briefly, 5 µL of each primer (2 nM) was added to a 48-well plate, along with 15 µL of a master mix containing 20 ng cDNA and 4 µL 5× HOTFIRE Pool^®^ EvaGreen^®^ qPCR Mix (Promega, Madison, WI, USA). The assays were run under the cycling conditions recommended by the manufacturer. Relative expression was analyzed using the model proposed by Pfaffl et al. [61]. Primers for ribosomal protein genes L13a (*RpL13a*) L4 (*RpL4*) and S29 (*Rps29*) were included in each plate and these reactions were used as references for mRNA normalization.

### 4.11. Serum Cytokine Quantification

Th1 cytokines, tumor necrosis factor alpha (TNF-α), interleukin-2 (IL-2), and interferon-gamma (IFN-γ) and Th2 cytokines, IL-4 and IL-5, were quantified simultaneously using a BD™ Mouse Th1/Th2 Cytokine Cytometric Bead Array (CBA) kit and BD™ CBA FCAP array software (BD Biosciences, San Jose, CA, USA), following the manufacturer’s instructions. The BD^TM^ FACSCanto II system (Becton Dickinson) was calibrated with setup beads, and 3000 events were acquired for each sample. Individual cytokine concentration ratios were indicated by their fluorescent intensities.

### 4.12. Statistical Analysis

Data were analyzed using GraphPad Prism version 7.0a (GraphPad Software, San Diego, CA, USA). Data were presented as the mean ± standard deviation (SD). Differences between groups involving continuous variables were evaluated by one-way analysis of variance (ANOVA). A two-way ANOVA was carried out in order to evaluate the influence of food consumption, treatments or their interaction on the body weight of the animals under study. Statistical significance was set at *p* < 0.05.

## Abbreviation

LDLLow-density lipoprotein*Ldlr*-/-LDL receptor nullSMCSmooth muscle cellTNF-αTumor necrosis factor alphaILInterleukinIFN-γInterferon-gammaCcl2
*chemokine (C-C motif) ligand 2*
TeffEffector T cellTregRegularoty T cellCD25Interleukin-2 receptor alpha chainFoxP3 transcription element fork-head box P3PDGProdigiosin-like pigmentUPGUndecylprodigiosinCTLCytolytic T lymphocytesNONitric oxideiNOSInducible nitric oxide synthaseLPSLipopolysaccharideMAPKmitogen-activated protein kinaseJNKc-Jun N-terminal kinaseNF-κBnuclear factor-kappa BCOX-2cyclooxygenase-2Th1T helper cell 1HUVECHuman umbilical vascular endothelial cellPCRPolymerase chain reactionVcam-1vascular cell adhesion molecule 1Aoc3amine oxidase, copper containing 3Pecam-1platelet endothelial cell adhesion moleculeAbca1ATP-binding cassette transporter A

## Figures and Tables

**Table 1:** Lipid profile of C57 atherosclerotic mice, treated with prodigiosin and undecylprodigiosin for 28 days.

Group	TC (mg/dL)	HDL-C (mg/dL)	LDL-C (mg/dL)	TG (mg/dL)
Control	1585 ± 400	35.2 ± 6.1	1376 ± 100.5	435.0 ± 114.7
P50	1480 ± 193	28.7 ± 6.7	1367 ± 208.4	423.3 ± 90.0
P500	1735 ± 238	22.8 ± 8.6	1602 ± 193.7	402.0 ± 64.2
U50	1653 ± 326	35.3 ± 7.4	1535 ± 371.7	411.7 ± 140.3
U500	1616 ± 391	28.3 ± 11.9	1435 ± 459.0	435.7 ± 68.0

TC, total cholesterol; HDL-C, high-density lipoprotein cholesterol; LDL-C, low-density lipoprotein cholesterol; TG, triglycerides; P50, 50 µg/Kg/day PG; P500, 500 µg/Kg/day PG; U50, 50 µg/Kg/day UPG; U500, 500 µg/Kg/day UPG. The analyte content was measured in all individuals of each group.
